# An intervention to support stroke survivors and their carers in the longer term (LoTS2Care): study protocol for a cluster randomised controlled feasibility trial

**DOI:** 10.1186/s13063-018-2669-5

**Published:** 2018-06-11

**Authors:** Anne Forster, Suzanne Hartley, Lorna Barnard, Seline Ozer, Natasha Hardicre, Tom Crocker, Marie Fletcher, Lauren Moreau, Ross Atkinson, Claire Hulme, Ivana Holloway, Laetitia Schmitt, Allan House, Jenny Hewison, Gillian Richardson, Amanda Farrin, on behalf of the LoTS2Care Programme Management Group

**Affiliations:** 1 Academic Unit of Elderly Care and Rehabilitation, Leeds Institute of Health Sciences, University of Leeds, Leeds, UK; 2 Academic Unit of Elderly Care and Rehabilitation, Bradford Teaching Hospitals NHS Foundation Trust, Bradford, UK; 3 Clinical Trials Research Unit, Leeds Institute of Clinical Trials Research, University of Leeds, Leeds, UK; 4 Academic Unit of Health Economics, Leeds Institute of Health Sciences, University of Leeds, Leeds, UK; 5 Leeds Institute of Health Sciences, University of Leeds, Leeds, UK; 6 Centre for Health Services Research, Leeds Institute of Health Sciences, University of Leeds, Leeds, UK; 7 Leeds Institute of Cardiovascular and Metabolic Medicine, University of Leeds, Leeds, UK

**Keywords:** Stroke, Longer term, Feasibility trial, Community, Study within a trial (SWAT), Facilitated self-management, Complex intervention, Cluster trial

## Abstract

**Background:**

Despite the evidence that many stroke survivors report longer term unmet needs, the provision of longer term care is limited. To address this, we are conducting a programme of research to develop an evidence-based and replicable longer term care strategy. The developed complex intervention (named *New Start*), which includes needs identification, exploration of social networks and components of problem solving and self-management, was designed to improve quality of life by addressing unmet needs and increasing participation.

**Methods/Design:**

A multicentre, cluster randomised controlled feasibility trial designed to inform the design of a possible future definitive cluster randomised controlled trial (cRCT) and explore the potential clinical and cost-effectiveness of New Start.

Ten stroke services across the UK will be randomised on a 1:1 basis either to implement New Start or continue with usual care only. New Start will be delivered by trained facilitators and will be offered to all stroke survivors within the services allocated to the intervention arm. Stroke survivors will be eligible for the trial if they are 4–6 months post-stroke and residing in the community. Carers (if available) will also be invited to take part. Invitation to participate will be initiated by post and outcome measures will be collected via postal questionnaires at 3, 6 and 9 months after recruitment. Outcome data relating to perceived health and disability, wellbeing and quality of life as well as unmet needs will be collected. A ‘study within a trial’ (SWAT) is planned to determine the most acceptable format in which to provide the postal questionnaires. Details of health and social care service usage will also be collected to inform the economic evaluation. The feasibility of recruiting services and stroke survivors to the trial and of collecting postal outcomes will be assessed and the potential for effectiveness will be investigated. An embedded process evaluation (reported separately) will assess implementation fidelity and explore and clarify causal assumptions regarding implementation.

**Discussion:**

This feasibility trial with embedded process evaluation will allow us to gather important and detailed data regarding methodological and implementation issues to inform the design of a possible future definitive cRCT of this complex intervention.

**Trial Registration:**

ISRCTN38920246. Registered 22 June 2016.

**Electronic supplementary material:**

The online version of this article (10.1186/s13063-018-2669-5) contains supplementary material, which is available to authorized users.

## Background

Stroke remains a major illness, with over 1 million people living in the United Kingdom who have had a stroke and over 100,000 people who suffer a stroke each year. Almost two-thirds of stroke survivors leave hospital with a disability [1]. The early stages of the stroke care pathway are becoming more prescribed (treatment in acute and rehabilitation stroke units), but despite policy recommendations, strategies for longer term care are not developed and longer term outcomes remain poor for many stroke survivors and their carers [2–4]. Many stroke survivors report unmet needs 1–5 years after stroke [5]. To address the limited provision of longer term stroke care, we have developed a new intervention designed to form part of a replicable ‘care strategy’ aimed at improving stroke survivors’ quality of life by addressing unmet needs and enhancing participation.

The developed intervention, called *New Start*, involves an assessment of stroke survivors’ needs and a review of their social networks at approximately 6 months post-stroke (purposely coinciding with national guidelines for a 6-month review of health and social care needs [6]). An open process then seeks to enable stroke survivors to address their identified needs and includes components of problem solving and self-management. The intervention is delivered by trained staff we call the New Start facilitator(s).

Herein, we describe the protocol for a feasibility trial intended to inform the design of a possible future definitive randomised controlled trial (RCT) and explore the potential clinical and cost-effectiveness of the New Start intervention.

## Methods/design

### Aims and objectives

We aim to conduct a pragmatic, multicentre, cluster randomised controlled feasibility trial of the New Start intervention versus usual care practice. The key objectives of this trial relate to gathering data to inform the feasibility and acceptability of implementing a future definitive cluster randomised controlled trial (cRCT); these are listed below and described in more detail in Additional file 1.

1. Stroke service recruitment methods and uptake
2. Stroke survivor recruitment methods and uptake
3. Intervention implementation and delivery
4. Definition of usual care
5. Assessment of outcome measures, potential for effectiveness and data gathering to assess the intracluster correlation coefficient (ICC)
6. Assessment of cost and cost-effectiveness
7. Safety

**Fig. 1:**
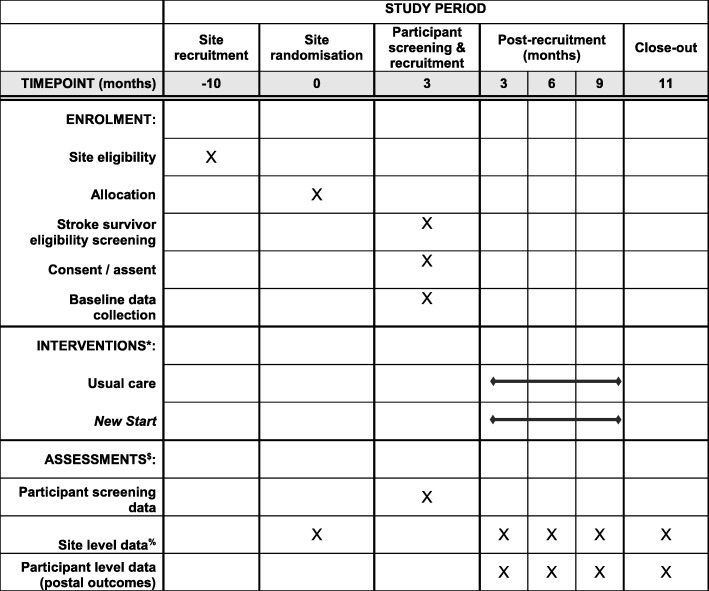
SPIRIT figure. *Interventions delivered at site level for duration of trial until at least 2 months after participant recruitment approach has ended. ^$^Further detail is provided in Table 1. ^%^ Timepoints are post-randomisation

Figure 1 illustrates the timing of all trial processes (Additional file 2).

### Setting

#### Stroke service eligibility criteria

The study will be conducted in 10 United Kingdom (UK) National Health Service (NHS) stroke services. A stroke service is defined by fulfilling a set of criteria, namely (1) encompasses the stroke pathway from secondary (hospital) care to primary/community-based care over a defined geographical area within the UK and (2) includes a stroke unit (given that treatment in a stroke unit is the recommended care pathway for all patients after a stroke [6, 7]). Stroke services will be included in this feasibility trial if they agree to try and establish a robust mechanism to identify all stroke survivors at 4–6 months post-stroke and have the facilities and capacity to deliver the New Start intervention, including training (i.e. staff available to undertake training and provide face-to-face contact with community-based stroke survivors at least 6 months post-stroke). Stroke services that have previously participated in research leading to the development of the intervention or that are currently or intending to implement a service comparable with the New Start intervention (e.g. a self-management focussed approach) within the proposed duration of the study will not be eligible.

All participating sites will be requested to complete a site survey documenting current service provision (including number of patients offered their service in the last 6 months) when expressing interest in the study, as well as at pre-randomisation (baseline), pre-recruitment and during recruitment (i.e. three monthly until the end of the follow-up period). This survey will allow capture of usual care and assessment and documentation of any change in stroke service provision (aside from the New Start intervention) during the course of the trial. The baseline survey will inform stratification factors for randomisation. Sentinel Stroke National Audit Programme (SSNAP) data will be reviewed to assess usual patient throughput in each service to enable comparison with recruitment rates during the trial.

### Stroke survivor eligibility

Stroke survivors will be eligible for inclusion in the study if they (1) are at least 4 months and not more than 6 months since confirmed primary diagnosis of new stroke; (2) are residing in the community (i.e. not in a nursing or residential care home); (3) are included within the defined population covered by the stroke service; (4) provide informed consent or consultee declaration; and (5) return a completed baseline questionnaire.

It is unknown whether the New Start intervention can be delivered to stroke survivors with other (specific) co-morbidities. Therefore, no exclusion criteria will be applied and reasons for not being offered or provided the New Start intervention at 6 months post-stroke (for those in a service so randomised) will be documented and used to inform eligibility criteria for a definitive trial.

### Carer eligibility

Carer involvement is not a requirement for stroke survivor inclusion in this trial. All carers identified by the stroke survivor as the main informal caregiver who provides the stroke survivor with support a minimum of once per week and who provide informed consent (i.e. implied via return of completed baseline questionnaire) will be eligible for inclusion in the study.

### Unit of randomisation

Cluster randomisation will reduce the likelihood of between-group treatment contamination. The New Start intervention aims to impact on skills, knowledge and clinical practice, and therefore the risk of contamination will be high if randomisation is at the individual stroke survivor level. All identified stroke survivors will be offered the opportunity to receive the allocated treatment and therefore it is not possible to use the patient as the unit of randomisation. Allocation of stroke services (clusters) to intervention or control will be undertaken independently by the statistician at the Clinical Trials Research Unit (CTRU). Stroke services will be allocated on a 1:1 basis to either implement the New Start intervention or continue with usual care only, using minimisation with a random element. Minimisation factors will be the number of stroke survivors seen by community teams per annum (above and below the median across all recruited services) and whether recruitment and intervention are delivered at separate Trusts (Yes/ No).

### Intervention

Intervention development was based on the principles of the Medical Research Council (MRC) framework for the development of complex interventions [8]. Details of the development of the intervention will be published separately. Briefly, a series of systematic literature reviews helped us identify the key unmet needs in the target population and interventions that had previously been evaluated. A national survey captured existing service models whereas focus group discussions and interviews with service users and carers provided important insights into the form and content of an acceptable intervention. The intervention was then developed using a structured process that involved exploration of this primary data to gain an understanding of the needs of stroke survivors and carers as well as potential outcomes of the intervention, and convening a multi-disciplinary expert group to co-design the intervention, in conjunction with the programme management team and consumer reference advisory group. We used a range of approaches to develop the intervention plan, including techniques derived from problem structuring, priority-setting and knowledge mobilisation. The emerging intervention and trial methodology were refined in a pilot study in three stroke services.

The resulting intervention, called New Start, is a programme of facilitated self-management that aims to improve longer term outcomes for stroke survivors. The emphasis is on improving quality of life by addressing unmet needs and enhancing participation, and is delivered in one-to-one meetings that are guided by principles of patient centredness, empowerment for patients and carers, and open availability for feedback.

The intervention involves an initial assessment of stroke survivors’ needs and a review of their social networks. An open process facilitated by trained staff then seeks to enable survivors to address their identified needs and use components of problem solving – prioritising needs, action planning, goal setting, and reviewing as well as self-management. At each stage, participants are encouraged to see those in their social network as resources to help in this process. Intervention delivery will be recorded by the New Start facilitators on purposely designed activity records.

#### New Start facilitator and training

The intervention is delivered by trained staff we call the New Start facilitator(s). All activities relating to delivery of the training and intervention implementation will be fully documented to provide a record of fidelity in terms of study design and provider training, informed by the Borelli framework [9].

Stroke services randomised to the intervention arm will identify New Start facilitator(s) who will be trained in the intervention. The number of facilitators required in each stroke service will depend on the size of the service and available staff. However, it is anticipated that approximately two facilitators will be trained at each stroke service. It is anticipated that the New Start facilitators will have experience in one of the following roles: Nurse, Physiotherapist, Occupational Therapist, Health and Wellbeing Practitioner, or other Allied Health Professional training, and will have stroke-specific knowledge and/or training.

New Start training will entail attending a structured training course involving face-to-face training supported by additional written materials. During the training, facilitators will learn relevant theory about a self-management approach and communication skills, as well as specific details about the intervention and how to deliver it to stroke survivors.

The New Start facilitators will be assessed for competency in the delivery of the New Start intervention, through review of patient activity records, reflective reports, interviews and observation, approximately 16 weeks after completing the initial training course (or earlier if appropriate).

#### Delivery and monitoring of the New Start intervention

The New Start intervention will be offered to all stroke survivors within the stroke services allocated to the intervention arm. Compliance with the New Start intervention will be monitored throughout the trial via observations and regular collection of activity records to assess adherence, to understand whether the New Start facilitators deliver the intervention in accordance with training and ‘as intended’. A parallel process evaluation (to be published separately) will enable evaluation of the training package and implementation process.

### Usual care

Stroke services randomised to the usual care control group will continue to deliver care as determined by local policy and practices. Our earlier survey work demonstrated that the provision of longer term support for stroke survivors is variable across the country. A total of 203 services in England were surveyed and, out of those who responded (*n* = 115), it was found that 70% commission community stroke services beyond 6 months. We plan to systematically collect data on usual care provision in all participating services.

### Recruitment and consent

Screening and approaching stroke survivors for inclusion in the trial will commence at all stroke services approximately 12 weeks post-randomisation to allow time for the New Start facilitators to be trained in the intervention.

#### Identification and screening of stroke survivors

Methods to identify stroke survivors and their carers (if available) by site staff may vary between sites, but it is anticipated that identification will be through routine data – this may include locally held registers or the SSNAP data. The proposed identification mechanism will be clarified with the local sites to ensure that the methods are inclusive of all stroke survivors and not a select sub-sample (e.g. only stroke survivors referred to an early supported-discharge team) and can be undertaken independent of the staff delivering any intervention to participants (in either arm) in order to reduce the potential for selection bias from differential recruitment, a particular concern in cRCTs [10].

A consecutive sample of stroke survivors will be identified and screened for eligibility on survival status and place of residence by site recruitment staff at participating stroke services.

The New Start facilitators in the intervention arm will have access to a list of all consecutive post-stroke survivors in their defined catchment area.

#### Approach and informed consent

Recruitment procedures have been designed to be as flexible as possible in order to optimise engagement with this study. Eligible stroke survivors will be initially approached via a letter inviting them to take part in the trial. Site staff will have the option to chase up any non-responders via telephone. Stroke survivors who express interest in the study are asked to indicate a preference for completion of baseline questionnaires, either face-to-face or by post. They can also gain further information about the study by telephone.

**Fig. 2:**
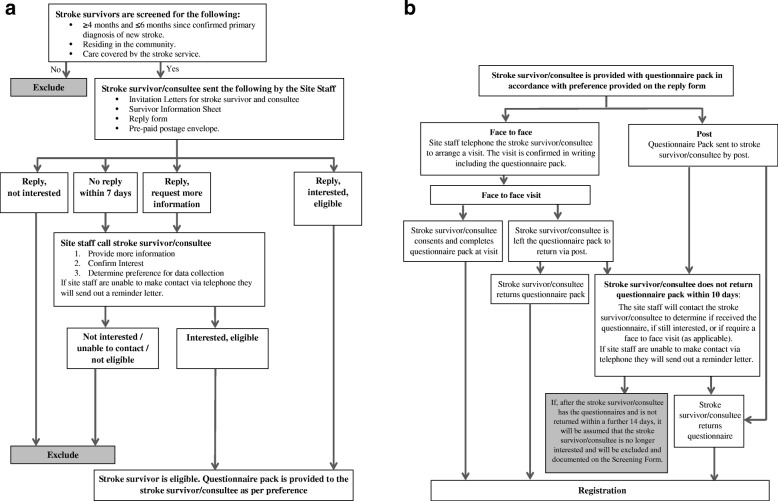
**a** Participant identification flow diagram. **b** Baseline consent and questionnaire completion flow diagram

Those stroke survivors interested in taking part will be provided with a baseline questionnaire pack, which will include a consent form, by their preferred method. In addition to consent to complete outcome assessments, participants will be asked if they consent to permit access to their electronic healthcare records. Figure 2 illustrates the participant identification and baseline consent and questionnaire completion processes.

#### Carer consent

Carer information packs, including a baseline questionnaire booklet, will be provided to the stroke survivor with their baseline questionnaire pack with a request to pass on to a carer if appropriate. Consent of the carer will be implied if the baseline carer questionnaire is returned completed.

#### Lack of capacity

To ensure that the trial population is representative of the clinical stroke population and inclusive of stroke survivors with cognitive impairment (including receptive, comprehension or language difficulties), procedures for Consultee Declaration will be implemented in compliance with the Mental Capacity Act 2005.

As it may not be clear at the time of approach whether the stroke survivor may lack capacity to consent for themselves, an invitation letter for a potential consultee, the stroke survivor’s family member, carer or friend, will be enclosed with all invitation letters posted out. All stroke survivor/consultee and carer information and consent documentation can be found in Additional file 3.

#### Study withdrawal

Stroke survivors (or where stroke survivors lack capacity, their consultee) and carers will be free to withdraw consent and leave the study at any time without giving reasons and without their care being affected. We will clarify whether they are withdrawing from the completion of questionnaires, access to health and social care records or both. Previously collected data will still be used in the analyses (unless consent has been withdrawn). Where possible, the reason for stroke survivor and/or carer withdrawal will be collected.

### Data collection

Data will be collected at the level of the service (including staff) as well as from individual consenting participants at baseline and at 3, 6 and 9 months post recruitment. Personal information will be held centrally, in accordance with consent, by the research team to facilitate follow-up contact, but will be stored and processed separately to all other data collected for the purposes of the trial.

#### Stroke survivor

Stroke survivor screening data will be recorded by site recruitment staff and will include, where possible, demographic characteristics (age, sex and ethnicity), hospital admission and discharge details, Modified Rankin Scale (mRS) score at discharge, National Institute for Health Stroke Scale (NIHSS) score at admission and availability of a carer. The reasons for ineligibility or for declining participation will also be recorded.

The baseline data will also be recorded by site recruitment staff and, in addition to the screening data, will include where possible: date of birth, NHS identification number, contact details, GP details, living arrangement details, carer details (if applicable), date of stroke, details on the level of impairment at recruitment (e.g. speech impairment, difficulties with communication) and preferred method(s) of contact.

#### Baseline and follow-up assessments

One of the aims of this trial is to assess the acceptability of the assessment methods and tools with a view to simplifying this for the planned definitive cRCT. Therefore, we have chosen not to test the use of outcome assessments that have been used successfully in previous stroke trials.

##### Stroke survivor

**Fig. 3:**
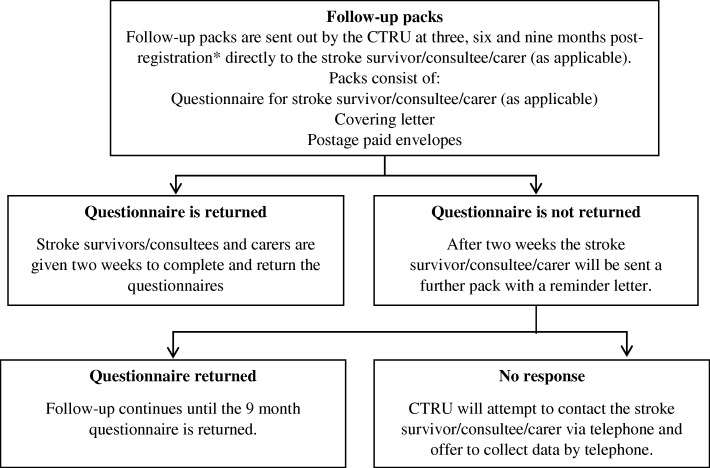
Follow-up flow diagram. *Prior to posting the follow-up pack the CTRU will confirm the stroke survivors’ and carers’ survival status and current address. Subject to consent and collection of phone number from the stroke survivor/consultee/carer; CTRU will send reminders to the stroke survivor/consultee/carer via Short Message Service (SMS) text message as a prompt to complete the questionnaires

Stroke survivor baseline assessments will be administered by the site staff either in person or by post. Stroke survivors will be followed up by the central trial team via postal questionnaires at 3, 6 and 9 months post recruitment. This will be supported by postal, telephone and text reminders if questionnaires are not returned within 2 weeks. Proxy completion of questionnaires by the stroke survivors’ carer/family/friend will be permitted. If outcome measures cannot be obtained by post, then telephone interviews may be conducted to collect the data to maximise return of data. Figure 3 illustrates the follow-up process.

The questionnaires will include the following validated outcome measures:

- World Health Organization Disability Assessment Schedule 2.0 36-item version, WHODAS 2.0 [11–13]: a generic measure of health and disability. It captures activity limitations and participation restrictions in six domains of life (understanding and communicating, getting around, self-care, getting along with people, life activities and participation in society) and a summary score.
- The Warwick-Edinburgh Mental Well-being Scale, WEMWBS [14–17]: measures feeling and functioning aspects of mental wellbeing or positive mental health.
- EQ-5D with 5 levels of severity for each of the 5 dimensions (EQ-5D-5L) [18–21]: a measure of health status or health-related quality of life (HRQoL) for clinical and economic evaluation. It measures problems in five domains (mobility, self care, usual activities, pain/discomfort, anxiety/depression) and self-rated health.
- ICEpop CAPability measure for Adults, ICECAP-A [22–27]: measures capability for five attributes of wellbeing (attachment, stability, achievement, enjoyment, autonomy) for use in economic evaluation.
- 13-item Short Form Patient Activation Measure Survey, PAM® survey [28, 29]: assesses the extent to which people feel engaged, with the knowledge, skills and confidence to manage a medical condition.
- Longer-term Unmet Needs after Stroke tool, LUNS [30]: a 22-item monitoring tool for identifying longer term unmet needs after stroke.
- Relevant questions adapted from the Millennium Survey of Poverty and Social Exclusion [31] and GP Patient Survey [32].

The questionnaires will also include a single overall life satisfaction question and a request for information about who completed the pack (the stroke survivor or a proxy) and how much help was provided. In addition, health, social care and voluntary or third sector service use will be collected together with costs to participants using a resource use questionnaire.

Information from stroke survivors’ health and social care records will also be collected from NHS Digital and other routine data sources, if relevant consent has been provided (subject to approval from the relevant data provider).

##### Carer

Carer baseline assessments will be administered to the carers in a similar manner to stroke survivors. Questionnaire packs will be provided to stroke survivors or their consultee with instructions to pass them to a carer if one exists. These questionnaires will be administered by post unless the carer is able to attend a face-to-face visit requested by the stroke survivor/consultee. Carers will be followed-up by the central trial team via postal questionnaires at 3, 6 and 9 months after stroke survivor recruitment. This will be supported by postal, telephone and text reminders if questionnaires are not returned within 2 weeks. If outcome measures cannot be completed by post, telephone interviews will be conducted by the central trial team to collect the data.

The questionnaires will include the Caregiver Burden Scale [33], which assesses the burden experienced by caregivers of people with chronic disability, EQ-5D-5L and ICECAP-A. Health, social care and voluntary or third sector service use will be collected together with costs to the carer using a resource use questionnaire.

A summary of the stroke survivor and carer assessment schedule is given in Table 1.

**Table 1:** Summary and timing of stroke survivor and carer assessments

Assessment	Screening	Baseline	Time-point (post registration)		
			3 months	6 months	9 months
Screening	X				
Eligibility		X			
Informed consent/Consultee declaration		X			
Baseline		X			
Registration		X			
Stroke Survivor Questionnaires					
Demographic details		X			
WHODAS 2.0		X		X	X
WEMWBS		X		X	X
PAM^®^ Survey		X	X	X	
EQ-5D-5L		X	X	X	X
ICECAP-A		X	X	X	X
LUNS		X			X
GP patient survey (two questions)		X	X	X	
Social questions		X	X	X	
Health and social care resource use (stroke survivor and provider reported)		X	X	X	X
Adverse events			X	X	X
Hospital admissions			X	X	X
Carer Questionnaires					
Demographic details (on each carer if applicable)		X	(X)	(X)	(X)
Caregiver Burden Scale		X	X	X	X
ICECAP-A		X	X	X	X
EQ-5D-5L		X	X	X	X
Health and social care resource use		X	X	X	X

*EQ-5D-5L* EQ-5D with 5 levels of severity for each of the 5 dimensions, *ICECAP-A* ICEpop CAPability measure for Adults, *LUNS* Longer-term Unmet Needs after Stroke tool, *PAM* Patient Activation Measure, *WEMWBS* Warwick-Edinburgh Mental Well-being Scale, *WHODAS* World Health Organization Disability Assessment Schedule

##### Method of administration

The questionnaires consist of a number of outcome measures alongside a resource use questionnaire. We would like to determine the most acceptable format in which to provide these questionnaires in order to maximise follow up rates for the future definitive trial. We therefore have planned a study within a trial (SWAT) [34] where we will randomise the method of delivery of the outcome assessments. This means that stroke survivors (and carers where available) will receive, at random, follow-up questionnaires in one of the following formats: one booklet (including all measures) or two (one including the outcome measures and the other with the resource use questionnaire). Randomisation will be undertaken independently by the CTRU just prior to the 6 months follow-up time point. Stroke survivors will be randomised on a 1:1 basis to receive the questionnaires in the formats indicated above at the six and nine month follow up time points (where applicable, the carer will receive the format allocated to the stroke survivor).

#### Data collection on care received

Details regarding usual care at each participating site will be captured by the site survey.

In addition, stroke service clinical staff in all participating sites will be asked to keep an activity record for each stroke survivor they offer/provide a service to six months after their stroke. Intervention specific activity records will also be completed by the facilitators delivering the New Start intervention; this will enable audit of the number of stroke survivors in receipt of the New Start intervention, as well as assessment of the adherence and fidelity of the intervention delivered.

NHS Digital data will also be used to provide information on hospital admissions and outpatient attendance received by each of the stroke survivors during the course of the trial (from time of recruitment to nine months post-recruitment).

#### Safety reporting

We will collect data on related and unexpected serious adverse events. Expected events such as death of a stroke survivor / carer or institutionalisation will also be recorded and will be collected from the date of consent until nine months post-recruitment. We are exploring the feasibility of using routine data for collecting this information.

### Blinding

Blinding in cluster trials is particularly problematic. We are implementing a range of strategies to try and maintain blinding where possible. The recruiting team will not be informed of the outcome of the cluster randomisation and therefore the treatment allocation for their site. This will allow us to minimise bias in the recruitment process.

In order to minimise any treatment bias, the New Start facilitators will not be informed of which of their patients are participating in the trial.

Where incidents of unblinding do occur, site staff (from recruitment and clinical teams) will be asked to inform the study team as soon as possible providing detailed information on the circumstances surrounding the incident. This will allow such incidents to be fully documented and reported on and this information will be used to inform the design of the future definitive trial.

### Process evaluation

A parallel process evaluation will be undertaken based on the MRC guidance for conducting process evaluations of complex interventions [8, 35] and Grant’s framework for process evaluations of cluster-randomised trials [36]. Process evaluation methods were subject to a separate ethics application and will be described in a separate paper. In brief summary here, the primary objectives of the process evaluation are to:

1. Assess implementation fidelity.
2. Explore and clarify causal assumptions regarding implementation.
3. Investigate the contextual factors associated with variations in intermediate outcomes between sites (e.g. levels of available resources, staff).
4. Explore the views, perceptions and acceptability of the intervention to facilitators, stroke survivors and carers.
5. Test and refine methods of data collection and interrogation in preparation for a process evaluation alongside a future effectiveness trial.

We also propose to explore with the recruitment team at each site the barriers and facilitators to the recruitment process.

### Economic evaluation

The economic evaluation will assess the cost effectiveness of New Start compared to usual care using both a within trial cost effectiveness analysis and a longer term cost effectiveness model. Both analyses will take a societal perspective including health and social care, voluntary and third sector providers together with costs to stroke survivors and their informal carers.

The analyses will use the Quality-Adjusted Life Year (QALY) outcome measure [37]. The estimation of QALYs is based on health-related quality of life (HRQoL) scores for health states and the EQ-5D-5L instrument will be used for this purpose [38, 39]. The EQ-5D-5L responses collected from stroke survivors and their carers will generate HRQoL scores using the UK population tariff. The potential for aggregating stroke survivor and carer QALYs within the economic analyses as well as alternative instruments to assess carers’ HRQoL will be explored. In addition, stroke survivors’ WHODAS 2.0 scores collected within the study will be mapped to their EQ-5D-5L scores, in order to add to the evidence base.

Data from earlier work-streams of the LoTS2Care programme will be used to identify mediators and moderators for consideration in the analyses. Data from the feasibility cRCT will further explore whether these variables could have a potential mediating or moderating effect on the economic evaluation.

Resource use (healthcare, social care, voluntary and third sector) together with out of pocket expenses and productivity losses will be collected by way of the stroke survivor and carer resource use questionnaires. The added value of NHS Digital and other routine data sources to collect healthcare resource use in any subsequent definitive cRCT will also be explored.

Unit costs for health service resources will be obtained from national sources such as the Personal Social Services Research Unit, the British National Formulary and the NHS Reference cost database. Where national unit costs are not available, the finance departments of Trusts participating in the study will be asked to provide local cost data. In line with recommendations from the National Institute for Health and Care Excellence, costs and outcomes will be discounted at 3.5% per annum [37].

### Sample size

We aim to recruit 200 stroke survivors (though the actual number of stroke survivors may be higher than this to account for uptake of the intervention and losses to follow-up) from 10 sites over a period of approximately 6 months. We judge it is achievable to recruit three to four stroke survivors from each site per month.

As this is a feasibility study, formal power calculations are not appropriate as clinical effectiveness is not being evaluated. The results generated from this study, including robust estimates for non-compliance and loss to follow-up rates in this patient group, will be used to inform the power calculation for a possible definitive study.

### Statistical analysis

Statistical analysis is the responsibility of the University of Leeds CTRU statistician, and a final statistical analysis plan will be written prior to any analysis being undertaken. All analyses and data summaries will be conducted on the intention-to-treat population, defined as all stroke survivors recruited, regardless of non-compliance with the protocol or withdrawal from the study. No formal interim or sub-group analyses are planned, and final analysis will take place when all available data have been received. The analysis will be focussed on descriptive statistics and confidence interval (CI) estimation rather than on formal hypothesis testing.

#### Estimation of stroke service and stroke survivor recruitment uptake

The number of stroke services expressing an interest in participation in the trial will be summarised. The number of services screened for selection together with reasons for non-selection will be presented.

The feasibility and success of the stroke survivor recruitment strategy will be evaluated by summarising the screening, eligibility, consent and approach processes, including the numbers of stroke survivors involved during each stage overall, by stroke service and by arm. Comparison with available national datasets (e.g. SSNAP) will be undertaken where applicable. Reasons for non-participation in the study will be summarised.

Retention of stroke services and participants (stroke survivors and carers) will be presented overall and by treatment arm. The number and timing of withdrawals of each of these from the trial, as well as reasons for withdrawal, will also be presented overall and by treatment arm. The number of New Start facilitators withdrawing from their role and the timing of and reasons for withdrawals (if available to us) will be summarised by stroke service and overall.

#### Assessment of intervention implementation and delivery

The feasibility of the intervention will be evaluated by summarising the numbers of New Start facilitators recruited and assessed as competent, by summarising the number of stroke services and New Start facilitators adhering to the intervention, and by summarising the number of stroke survivors completing the intervention by services and overall. The number of stroke services demonstrating adherence to the intervention delivery will be evaluated by review of the activity records. The embedded process evaluation will assess the intervention delivery in more detail.

#### Assessment of usual care

Returned site surveys and activity records will be reviewed and used to guide a definition of routine care for use in a future definitive trial.

#### Assessment of outcome measures, potential for effectiveness and data gathering to assess the ICC

The feasibility and success of obtaining the outcome data will be assessed at each time point by summarising the proportion of stroke survivors with available self-reported outcome data by site, treatment arm and overall. Follow-up rates and levels of missing data will similarly demonstrate the acceptability of the outcome measures. Levels of missing self-reported outcome data, both at the individual item level and for entire outcome measures, will be reported at each time point overall and by treatment arm. Acceptability of methods for obtaining the outcome data by post, face-to-face or over the phone and burden of collecting all questionnaires will also be summarised at each time point, by treatment arm and overall. Acceptability of the format of the questionnaires (one questionnaire incorporating all outcome measures and health resource use or two separate questionnaires, one with the outcome measures and the other with the health resource use alone) will also be summarised at each time point, by treatment arm and overall.

Acceptability, availability of and completion rates for routine data collection will be presented by site, treatment arm and overall. Stroke survivors’ use of NHS services and the number of deaths will be summarised from routine data and reported by stroke service, treatment arm and overall.

Eligibility and recruitment rates, along with the number of stroke survivors successfully followed up, will also help inform the sample size by assessing the feasibility of recruitment for the definitive trial.

Variation around the ICC estimate is likely to be large and we will estimate the ICC after controlling for covariates and constructing CIs to obtain a range of plausible estimates, from which a variety of sample sizes for the definitive trial can be calculated. As we may not be able to obtain a very precise estimate from our data, we will also compare our estimated ICC with those from other published studies. We will summarise outcome measures, predictive and process variables by point estimates, variability estimates and range of CIs (e.g. 95%, 67% and 51%) by randomised group at each time point.

In order to inform the sample size calculation for the definitive trial and determine potential for effectiveness, we will calculate scores for each outcome and differences in outcomes by treatment arm together with corresponding 95% CIs.

#### Assessment of cost and cost-effectiveness

The within trial cost effectiveness analysis will present an incremental cost effectiveness ratio (ICER) and the net monetary benefit (NMB). Missing data will be accounted for in line with current recommended methods and uncertainty using the bootstrapping method [40]. For the longer term cost effectiveness, a decision model will be developed and the results presented as an ICER and NMB. A value of information analysis will be undertaken in order to inform the design of any subsequent RCT. Missing data will use current recommended methods and sensitivity analysis using the Monte Carlo simulations will account for uncertainty [41, 42].

#### Safety

All safety reports will be independently reported and reviewed by the Programme Steering Committee (PSC).

### Criteria for continuation to the definitive RCT

Criteria for progression to a definitive RCT are based on recruitment, follow-up, and intervention implementation and delivery. Suggested progression criteria are detailed in Table 2 and are based on a traffic-light system of green (go), amber (review) and red (stop). The criteria are to be agreed by the independent PSC and funder.

**Table 2:** Criteria for continuation to the definitive RCT

Criteria	Green	Amber	Red
Average recruitment of participants per site over 6 months	≥ 20 (range 12–30)	< 20 but ≥ 10	< 10
Return of follow-up questionnaires	≥ 75%	< 75% but ≥ 60%	< 60%
Intervention training	At least two members of staff from each stroke service attending training days and assessed as competent		
Intervention delivery (% of recruited stroke survivors offered at least one session of the intervention)	≥ 75%	< 75% but ≥ 50%	< 50%
Intervention implementation (% of stroke services deemed competent and went on to deliver the intervention and recruited participants)	≥ 80% (i.e. 4 services)	60% (i.e. 3 services)	< 60% (i.e. 2 or less services)

### Data monitoring

Overall trial supervision is provided by the PSC, with an independent Chair as well as patient and public involvement representation. For a feasibility study of this nature and duration, a separate Data Monitoring and Ethics Committee is not required; rather, the PSC will adopt a safety monitoring role, with the constitution of a sub-committee to review safety issues where this becomes necessary.

Data will be monitored for quality and completeness by the CTRU. Missing data, except for individual data items collected via the postal questionnaires, will be chased until they are received, confirmed as not available, or when the trial is at analysis. Reminders will be sent to participants if postal questionnaires are not returned on time.

### Trial organisation and administration

This feasibility trial is the final study in a 5-year Programme Grant for Applied Research (grant RP-PG-0611-20010) funded by the National Institute for Health Research. The trial is sponsored by the Bradford Teaching Hospitals NHS Foundation Trust and is co-ordinated by the Academic Unit of Elderly Care and Rehabilitation (Bradford Teaching Hospitals NHS Foundation Trust and University of Leeds) and the CTRU (Leeds Institute of Clinical Trials Research, University of Leeds). The Trial Management Group is comprised of the Chief Investigator, key co-applicants, research fellows experienced in qualitative research and key members of the project delivery team at the CTRU.

The study is adopted by the UK Clinical Research Network and is supported in part by its trained research staff. The trial is registered (ISRCTN38920246). The trial will be conducted in accordance with the principles of Good Clinical Practice in clinical trials, as applicable under UK regulations, the NHS Research Governance Framework, and through adherence to CTRU standard operating procedures. Ethical approval has been obtained through the UK National Research Ethics Service (16/YH/0068).

### Dissemination

The results of the study will be published in peer-reviewed publications and will be presented at relevant national and international conferences. We will work with the patient and public involvement representatives to develop lay reports to disseminate research findings to patient groups and the clinical staff at participating sites. Authorship will be agreed in accordance with the LoTS2Care Programme publication policy and in line with International Committee of Medical Journal Editors (ICMJE) recommendations.

## Discussion

Despite the evidence that many stroke survivors report unmet needs, the evidence base for longer term stroke care for stroke survivors and their carers is limited. To address this, we are conducting a programme of research to develop and evaluate an evidence-based and replicable longer term care strategy. We have developed the New Start intervention, which aims to improve stroke survivors’ quality of life by addressing unmet needs and increasing participation.

This feasibility trial has been designed to inform the design of a possible future definitive cRCT and explore the potential clinical and cost-effectiveness of the New Start intervention. Due to the complex nature of the intervention and the design of the trial, we anticipate some critical implementation challenges, as described below.

### Recruitment

Recruitment of stroke survivors to research undertaken after the first weeks following the stroke is difficult as there is no easily accessible method of identification for clinical or research staff following discharge from hospital. Here, we are testing an approach which we hope may be replicable for other similar studies in the future.

### Service need

Whilst there is evidence that longer term outcome for people after stroke is poor, this may be from select patient groups. We are aiming to offer an intervention to all stroke survivors (at 4–6 months after their stroke). This will enable us, for the first time, to gain greater insights into the expressed need for longer term follow-up services from a wide group of stroke survivors, thus providing invaluable information to inform service provision.

### Implementation of the New Start intervention

A key aspect of this study is to implement and sustain a novel and complex intervention across different stroke service configurations. The embedded process evaluation will allow us to monitor adherence to the intervention and enable us to explore any barriers or enablers to the implementation of New Start to optimise implementation in a future definitive trial.

### Retention

The study involves follow-up of participants over a period of 9 months. Retention of participants over such a period of time may be an issue. We aim to minimise the risk of substantial losses to follow-up by performing postal, telephone and text reminders to chase up missing questionnaires and conducting telephone interviews in the event that outcome measures cannot be obtained by post.

This feasibility trial will enable us to identify any important methodological issues, assess stroke patients’ needs and intervention implementation, and collect useful data on recruitment uptake and outcome measure feasibility that will inform the design and development of a possible future definitive RCT, which could change the way in which longer term care is provided to stroke survivors.

### Trial status

The study commenced recruitment of stroke services in December 2015, and recruitment of stroke survivors and carers in January 2017. The study is projected to complete recruitment by end of November 2017.

## Additional files

Additional file 1: Detailed study objectives. This document includes further details on the study objectives. (DOCX 25 kb)

Additional file 2: SPIRIT checklist. This document includes the completed SPIRIT checklist. (DOC 123 kb)

Additional file 3: LoTS2Care trial information and consent documentation. This document includes all information sheets and consent forms provided to the stroke survivors and carers involved in the trial. (PDF 340 kb)

## Abbreviations

CI: confidence interval
cRCT: cluster randomised controlled trial
CTRU: Clinical Trials Research Unit
EQ-5D-5L: EQ-5D with 5 levels of severity for each of the 5 dimensions
HRQoL: health-related quality of life
ICC: intracluster correlation coefficient
ICECAP-A: ICEpop CAPability measure for Adults
ICER: Incremental Cost Effectiveness Ratio
MRC: Medical Research Council
NHS: National Health Service
NMB: Net Monetary Benefit
PSC: Programme Steering Committee
QALY: quality-adjusted life year
RCT: randomised controlled trial
REC: Research Ethics Committee
SSNAP: Sentinel Stroke National Audit Programme
UK: United Kingdom
WEMWBS: Warwick-Edinburgh Mental Well-being Scale
WHODAS: World Health Organization Disability Assessment Schedule
