# Supplementary material for: An intervention to support stroke survivors and their carers in the longer term (LoTS2Care): study protocol for a cluster randomised controlled feasibility trial
Source: Trials. 2018 Jun 11;19:317. doi: 10.1186/s13063-018-2669-5 (PMC5996505; doi:10.1186/s13063-018-2669-5)
Supplement: Supplementary file 1 — Detailed study objectives. This document includes further details on the study objectives. (DOCX 25 kb) [file 13063_2018_2669_MOESM1_ESM.docx]

**Detailed study objectives**

1. ***Stroke service recruitment methods and uptake***
2. To explore the number of stroke services screened and identified as eligible, in order to provide evidence of number of eligible services potentially available to meet the sample size requirements of the definitive trial.
3. ***Stroke survivor recruitment methods and uptake***
4. To assess the method used to identify stroke survivors at approximately six months post-stroke to inform the optimal recruitment process.
5. To assess the number of stroke survivors screened, identified as eligible and for whom informed consent/consultee declaration can be obtained to provide evidence of sufficient numbers available to meet the sample size requirements for a definitive trial.
6. To evaluate whether there is potential for selection bias.
7. ***Intervention implementation and delivery***
8. To assess whether it is feasible to successfully recruit and train New Start Facilitators to deliver the New Start intervention as measured by the completion of training and achievement of competency by an appropriate number of New Start Facilitators at each service randomised to the intervention.
9. To assess adherence to the intervention, by the New Start Facilitators and the stroke survivor(s).
10. To explore barriers and enablers to the implementation of the New Start intervention to optimise implementation in the definitive trial.
11. To explore stroke survivors’, carers’ and New Start Facilitators’ views of the New Start care strategy to inform refinement for the definitive trial.
12. ***Definition of usual care (UC)***
13. To characterise the range of UC across the participating stroke services.
14. ***Assessment of outcome measures & potential for effectiveness***
15. To assess the appropriateness of outcome measures (acceptable levels of questionnaire completion, overall follow-up rates at each time point and each mode of administration (via post, postal reminder, text and telephone reminder, telephone interviews and home visits)) to inform the choice of primary and secondary outcomes.
16. To assess the feasibility of use of routine data to collect secondary outcome data (e.g. hospital readmissions, death).
17. To assess levels and variability of missing data for the self-reported questionnaires at the scale and item level at baseline, three, six and nine months post-registration.
18. To assess variability of outcomes at baseline, three, six and nine months post-registration.
19. To assess the intracluster correlation coefficient (ICC).
20. ***Assessment of cost and cost-effectiveness***
21. To assess the feasibility and methods of collecting resource and outcome data, including use of routine data sources, for a future large scale cost-effectiveness analyses (CEA) in the definitive trial.
22. To estimate costs of implementing the intervention.
23. ***Safety***
24. To record relevant adverse events (AE) (e.g. hospital admissions, institutionalisation, death) and to confirm how best to collect these.
