# Supplementary material for: An intervention to support stroke survivors and their carers in the longer term (LoTS2Care): study protocol for a cluster randomised controlled feasibility trial
Source: Trials. 2018 Jun 11;19:317. doi: 10.1186/s13063-018-2669-5 (PMC5996505; doi:10.1186/s13063-018-2669-5)
Supplement: Supplementary file 3 — LoTS2Care trial information and consent documentation. This document includes all information sheets and consent forms provided to the stroke survivors and carers involved in the trial. (PDF 340 kb) [file 13063_2018_2669_MOESM3_ESM.pdf]

Delete this line, and then print on Hospital headed paper

SURVIVOR INFORMATION SHEET

LoTS2Care

Improving Longer Term Stroke Care

A longer-term care strategy for stroke – a feasibility study.

A large-print version of this sheet is available on request.

Invitation to take part in a research study

- We are inviting you to take part in a research project called the LoTS2Care study, which is taking place in your stroke service.
- Your Stroke Service has already agreed to take part.
- Before you decide whether to take part, we want you to understand what this involves. Please discuss it with your friends and relatives if you wish
- Please read this information leaflet carefully and take time to decide whether you would like to take part. Please ask if anything is unclear.
- You are free to decide whether or not to take part. If you choose not to it will not affect your care in any way.
- You can keep this information sheet to remind you about the study.

How to contact us:

If you have any questions about this study, please contact:

Name of Researcher: <<insert>>
Telephone: <<insert>>

What is the purpose of the study?

We are currently exploring how to develop services for people following a stroke. We have developed a new approach to addressing the needs of stroke survivors and their carers after a stroke. We are working with community stroke services to compare our approach to that usually provided.

We are particularly interested in how the different approaches affect stroke survivors' and carers' wellbeing. We will also be looking at costs involved to the health service. If this study shows that our approach may be helpful to stroke survivors and carers, it may be studied further in a bigger study where we hope to show that this approach is more beneficial to stroke survivors and their carers than that which is currently provided.

Why have I been invited to take part?

Your stroke service is involved in the study. We are inviting all stroke survivors who had a stroke in the last six months and are treated by your stroke service.

Do I have to take part?

No. This study is entirely voluntary. If you decide not to take part, you will still receive the same standard of care as those who do take part. If you do agree to take part you are free to withdraw at any time without giving a reason.

What will happen if I take part?

You will be asked to complete a reply form. This form will ask whether you would like to take part in this study and if so, how you would prefer to complete the questionnaires for the study.

You will be asked to complete four questionnaires over the next nine months. The first questionnaire will be provided to you when you let us know you want to join the study. The second, third and fourth questionnaires will be provided three, six and nine months later. The questions will be about how you feel and what you are able to do. You may find some of the questions quite sensitive; you do not have to answer any questions that you find distressing. We would like you to answer the questions as honestly as you can and return the questionnaire to us in the prepaid, addressed envelope that will be provided.

Someone, a friend or relative, can help you fill it in but you must think of your own responses. This way your answers will definitely be your own.

We will also send you newsletters to keep you updated on the study.

What else will happen if I take part?
The staff at your stroke service will look at your health care records to get information, for example, to see what care you have been receiving since you had a stroke or if you have seen a doctor recently.

We would also like to access you electronic health records from your hospital and your GP to obtain data which researchers think is important to your Stroke. To obtain this data we will use your identifiable information (for example, your NHS number, date of birth, postcode, sex and initials) to make sure that we have the right records. The University of Leeds will obtain this data from NHS Digital and your GP.

We will only collect information required for research purposes, this information will be kept securely by the research team and used only for research purposes.

With your permission, we may also need to telephone you or send you text messages relating only to this study.

Some participants, but not all, may also be asked if they would like to discuss their experiences of the treatment they have received with a researcher, you would be contacted separately about this.

If you are happy to take part please fill out the enclosed reply form and return it in the postage paid envelope provided.

What are the possible benefits and risks of taking part?
The information we get from this study will help us answer important questions to decide whether we should test the intervention in a bigger study. This could help us provide care and support to people who may have a stroke in the future and their carers.

We do not expect there will be any risk in taking part.

Will my taking part in this study be kept confidential?
Yes. All your details and answers to questions will be confidential. This information will be kept in a safe place in accordance with the Data Protection Act 1998.

Information about your Stroke and care you have received will be will be obtained from your medical notes by the research team. This and identifiable information, (such as your name, address and phone number, initials, data of birth, NHS number, sex and ethnicity) will be collected on a paper form by the research team at your hospital and sent (usually using standard post but in some cases by fax or email) to the University of

Leeds. Only some members of the research team will be able to look at this information. You will be given a unique number which will be used along with your date of birth and initials to identify you on each paper form.

No names will be used in any publications/reports. It is possible that the information you provide for this study may be shared with other research teams to answer new research questions in the future. If this happens your information would be entirely anonymous.

Your identifiable information will be shared with the data providers (e.g. NHS Digital) using a secure system to obtain relevant data from your electronic health records and to obtain information about your health status since your stroke.

Identifiable data will not be access or shared with any third parties outside the LoTS2Care Research team without your permission. All the data you give us will be stored for up to 10 years after the end of the study and then destroyed.

Your GP will be informed about your involvement in this study. We intend to contact your GP to verify your current contact details prior to sending out each questionnaire booklet.

What if I don't want to take part?
You don't have to take part if you don't want to and you don't have to give us a reason. It will not affect the care you receive.

If you are happy to take part now, but later decide to stop taking part, you can let us know by contacting us. We will stop collecting information about you but would like to use the data you provide in the study results.

What will happen to the results of the research study?
We aim to publish the results of this study in a reputable medical journal approximately one year after the study finishes. If you would like a short summary of the study please ask Professor Anne Forster (the Chief Investigator for the study, telephone number 01274 383406, email a.forster@leeds.ac.uk).

Who is involved in this research study?
This study is organised and run by the Academic Unit of Elderly Care and Rehabilitation (AUECR) at Bradford Royal Infirmary, the Leeds Institute of Clinical Trials Research (LICTR) and the Leeds Institute of Health Sciences (LIHS), at the University of Leeds. This study is funded by the Department of Health (National Institute for Health Research) and has been reviewed by Yorkshire & the Humber – Leeds East Research Ethics Committee.

There are no special compensation arrangements in place for this study. The normal NHS complaints mechanisms will still be available to you (if appropriate).

Thank you for taking the time to read this information sheet**. If you would like to know more, please contact** <<insert contact details>>

Delete this line and add Hospital header

A Longer Term Care Strategy for Stroke – A Feasibility Study (LoTS2Care)

STROKE SURVIVOR CONSENT FORM

Please read the statements below and sign on the next page to confirm you agree to take part in the above study:

1. I confirm that I have read and understood the Survivor Information Sheet, I have had opportunity to ask questions about the study and understand what is involved and I am happy to complete questionnaires about my abilities and experiences after stroke.
2. I understand that my participation in the study is voluntary and that I am free to withdraw at any time without my medical care or legal rights being affected.
3. I understand that even if I withdraw from the study, the data collected from me up to that point will be used in analysing the results of the study.
4. I understand that relevant sections of my medical/care records and data collected during the course of the study will be shared with members of the research team and may be looked at by responsible individuals from the NHS or regulatory authorities where it is relevant to my taking part in this research. I give permission for these individuals to have access to my records.
5. I understand that my personal identifiable data (e.g. initials, date of birth, NHS number, postcode and sex) will be collected and shared with the Health and Social Care Information Centre and the provider of my GP's clinical systems to obtain the electronic data held by my hospital and my GP.
6. I understand that my electronic data will be linked by the research team at the University of Leeds.
7. I agree to allow any information or results arising from this study to be used for further research upon the understanding that my identity will remain anonymous.
8. As part of this research I understand that I may be contacted to ask if I would like to discuss my experiences of the treatment I have received with a researcher.
9. I agree for my details and a copy of this consent form (which will include my name and date of birth) to be stored by the Research Office (at the University of Leeds) for the purposes of this study.
10. I agree that my GP, will be notified of my participation in this study. I agree to a copy of this Consent Form being sent to my GP.
11. I agree to take part in the above study.

The following point is OPTIONAL

Even if you agree to take part in this study, you do not have to agree to this statement

	Please tick (✓)
12. I agree to being contacted in the future about further research.	Yes ☐ No ☐

Stroke Survivor

Signature:

Name *(block capitals):*

Date:

THANK YOU

For researcher use only

Stroke survivor ID (Centre No, Trial No)

Stroke survivor date of birth (Day, Month, Year)

Stroke survivor initials

NHS/Hospital number

ISRCTN38920246

A Longer Term Care Strategy for Stroke – A Feasibility Study (LoTS2Care)

CONSULTEE DECLARATION FORM

Please read the statements below and sign on the next page to confirm you agree for your friend/relative to take part in the above study:

1. I confirm that I have read and understood the Survivor Information Sheet, I have had opportunity to ask questions about the study and understand what is involved. I believe my friend/relative would want to take part and I am happy to complete questionnaires about my friend/relative's abilities and experiences after stroke.
2. I understand that their taking part in the study is voluntary and that I can request that my friend/relative is withdrawn at any time without giving a reason and without his/her medical care or legal rights being affected.
3. I understand that even if my friend/relative is withdrawn, the information collected about him/her from me up to that point will be used in analysing the results of the study.
4. I understand that relevant sections of my friend/relative's medical/care records and data collected during the course of the study will be shared with members of the research team and may be looked at by responsible individuals from the NHS or regulatory authorities where it is relevant to him/her taking part in this research. In my opinion my relative/friend would be willing to give permission for these individuals to have access to his/her records.
5. I understand that my friend/relative's personal identifiable data (e.g. initials, date of birth, NHS number, postcode and sex) will be collected and shared with the Health and Social Care Information Centre and the provider of their GP's clinical systems to obtain the electronic data held by their hospital and their GP.
6. I understand that my friend/relative's electronic data will be linked by the research team at the University of Leeds.
7. I agree to allow any information or results arising from this study to be used for further research upon the understanding that my friend/relative's identity will remain anonymous.
8. As part of this research I understand that I may be contacted to ask if I would like to discuss my friend/relative's experiences of the treatment they have received with a researcher.
9. I agree for my friend/relative's details and a copy of this declaration (which includes my name) to be stored by the Research Office (at the University of Leeds) for the purposes of this study.
10. I agree that my friend/relative's GP, will be notified of his/her participation in this study. I agree to a copy of this declaration being sent to his/her GP.
11. In my opinion my friend/relative would have no objection to taking part in this study and I agree for him/her to take part.

The following point is OPTIONAL

Even if you agree to your friend/relative taking part in this study, you do not have to agree to this statement

	Please tick (✓) Yes	No
12. I agree to my friend/relative being contacted in the future about further research.	☐	☐

Consultee (on behalf of relative/friend who has had a stroke)

Signature:

Name *(block capitals):*

Date:

For researcher use only

Stroke survivor ID (Centre No | Trial No)

Stroke survivor date of birth (Day | Month | Year)

Stroke survivor initials

NHS/Hospital number

ISRCTN38920246

Delete this line, then print on Hospital headed paper

<<Insert Patient Name and Address>>

07 November 2016

A Longer Term Care Strategy for Stroke – A Feasibility Study (LoTS2Care)

ADDENDUM TO STROKE SURVIVOR INFORMATION SHEET AND INFORMED CONSENT DOCUMENT

Dear <<Insert Patient Title and Surname>>

Thank you for completing the questionnaires as part of the LoTS2Care study (A longer-term care strategy for stroke). In order to obtain a thorough understanding of the care you have received we would like to access you electronic health records from your hospital and your GP to obtain data which researchers think is important to your Stroke. To obtain this data we will use your identifiable information (for example, your NHS number, date of birth, postcode, sex and initials) to make sure that we have the right records. The University of Leeds will obtain this data from NHS Digital and your GP.

Your identifiable information will be shared with the data providers (e.g. NHS Digital) using a secure system to obtain relevant data from your electronic health records and to obtain information about your health status since your stroke.

As part of this research some participants, but not all, may also be asked if they would like to discuss their experiences of the treatment they have received with a researcher. If you are selected we will contact you separately about this. In addition, there might be further research in the future, please tell us if you would like us to let you know about any other research projects in the future.

We would also like to be able to use your data for future research.

We are therefore writing to you to ask for your permission to do the following:

- To access your medical/care records and information held and maintained by The Health and Social Care Information Centre (HSCIC) and the provider of your GP's clinical systems.
- To allow any information or results arising from this study to be used for further research but your identity will remain anonymous.
- To contact you in the future about any further research that may be completed.

Once you have had time to consider this letter, please read the statements below and tell us whether you agree or not by **ticking** the Yes/No box:

	Please tick (✓)	
I understand that my personal identifiable data (e.g. initials, date of birth, NHS number, postcode and sex) will be collected and shared with the Health and Social Care Information Centre and the provider of my GP's clinical systems to obtain the electronic data held by my hospital and my GP. I understand that my electronic data will be linked by the research team at the University of Leeds.	YES ☐	NO ☐
I agree to allow any information or results arising from this study to be used for further research upon the understanding that my identity will remain anonymous.	YES ☐	NO ☐
I agree to being contacted in the future about further research.	YES ☐	NO ☐

Please sign and date the form below before returning this letter in the postage paid envelope provided:

Signature: ____________________

Name *(block capitals)*: ____________________

Date: ____________________

If you have any queries about this letter or would like to discuss this study further, please contact the research team on <<insert contact details>>.

Thank you for your help with this important study, it is greatly appreciated.

Yours sincerely,

<<Name>>
<<Contact Details>>

To be completed by the researcher:

Participant ID: Centre No / Trial No

Delete this line, and then print on Hospital headed paper

CARER INFORMATION SHEET

A Longer Term Care Strategy for Stroke – A Feasibility Study (LoTS2Care)

A large-print version of this sheet is available on request.

Invitation to take part in a research study

- We are inviting you to take part in a research project called the LoTS2Care study, which is taking place in your friend/relative's stroke service.
- Your friend/relative's Stroke Service has already agreed to take part.
- Before you decide whether to take part, we want you to understand what this involves. Please discuss it with your friends and relatives if you wish.
- If you decide to participate, you will be required to complete four questionnaire booklets.
- Please read this information leaflet carefully and take time to decide whether you would like to take part. Please ask if anything is unclear.
- Consent for this study is implied. This means that if you return the first questionnaire we will assume that you are happy to take part in the study and for us to contact your GP.
- You are free to decide whether or not to take part. If you choose not to it will not affect your friend/relative's care in any way.
- You can keep this information sheet to remind you about the study.

How to contact us:

If you have any questions about this study, please contact:

Name of Researcher: <<insert>>
Telephone: <<insert>>

What is the purpose of the study?

We are currently exploring how to develop services for people following a stroke. We have developed a new approach to addressing the needs of stroke survivors and their carers after a stroke. We are working with community stroke services to compare our approach to that usually provided.

We are particularly interested in how the different approaches affect stroke survivors' and carers' wellbeing. We will also be looking at costs involved to the health service. If this study shows that our approach may be helpful to stroke survivors and carers, it may be studied further in a bigger study where we hope to show that this approach is more beneficial to stroke survivors and their carers than that which is currently provided.

Why have I been invited to take part?

The stroke service where your friend/relative receives their care is involved in the study. We are inviting all stroke survivors who had a stroke in the last six months and their carers to participate.

Do I have to take part?

No. This study is entirely voluntary. If you decide not to take part, you and your relative/friend will still receive the same standard of care as those who do take part. If you do agree to take part you are free to withdraw at any time without giving a reason.

What will happen if I take part?

A researcher will ask you some questions (e.g. your contact details) and you will be asked to complete four identical questionnaires over the next nine months. The first questionnaire will be provided to you when your friend/relative lets us know they want to join the study. If you return this completed questionnaire the second, third and fourth

questionnaires will be provided three, six and nine months later. The questions will be about how you feel and what you are able to do. You may find some of the questions quite sensitive; you do not have to answer any questions that you find distressing. We would like you to answer the questions as honestly as you can and return the questionnaire to us in the prepaid, addressed envelope that will be provided.

Someone, a friend or relative, can help you fill it in but you must think of your own responses. This way your answers will definitely be your own.

With your permission, we may also need to telephone you or send you text messages relating only to this study.

Some participants, but not all, may also be asked if they would like to discuss their experiences with a researcher, you would be contacted separately about this.

If you are happy to take part please fill in the enclosed questionnaire and reply form and return them in the postage paid envelope provided.

What are the possible benefits and risks of taking part?

The information we get from this study will help us answer important questions to decide whether we should test the intervention in a bigger study. This could help provide care and support to people who may have a stroke in the future and their carers.

We do not expect there will be any risk in taking part.

Will my taking part in this study be kept confidential?

Yes. All your details and answers to questions will be confidential. This information will be kept in a safe place in accordance with the Data Protection Act 1998. Any identifiable data, (such as your name and your contact details) will be removed from any information you provide and will be stored separately. Only some members of the research team will be able to look at this information. You will be given a unique number which will be used along with your date of birth and initials to identify you.

No names will be used in any publications/reports. It is possible that the information you provide for this study may be shared with other research teams to answer new research questions in the future. If this happens your information would be entirely anonymous.

Information needed for trial purposes will be collected on paper forms and sent (usually using standard Royal Mail post but in some cases by fax or email) to the University of Leeds.

Identifiable data will not be accessed or shared with any third parties outside the LoTS2Care Research team. All the data you give us will be stored for up to 10 years after the end of the study and then destroyed.

Your GP will be informed about your involvement in this study. We intend to contact your GP to verify your current contact details prior to sending out each questionnaire booklet. We will not ask your GP to provide us with any medical information about you.

What if I don't want to take part?
You don't have to take part if you don't want to and you don't have to give us a reason. It will not affect the care you or your friend/relative receives.

If you are happy to take part now, but later decide to stop taking part, you can let us know by contacting us. We will stop collecting information about you but would like to use the data you provide in the study results.

What will happen to the results of the research study?
We aim to publish the results of this study in a reputable medical journal approximately one year after the study finishes. If you would like a short summary of the study please ask Professor Anne Forster (the Chief Investigator for the study, telephone number 01274 383406, email a.forster@leeds.ac.uk).

Who is involved in this research study?
This study is being organised and run by the Academic Unit of Elderly Care and Rehabilitation (AUECR) at Bradford Royal Infirmary, the Leeds Institute of Clinical Trials Research (LICTR) and the Leeds Institute of Health Sciences (LIHS), at the University of Leeds. This study is funded by the Department of Health (National Institute for Health Research) and has been reviewed by Yorkshire & the Humber – Leeds East Research Ethics Committee.

There are no special compensation arrangements in place for this study. The normal NHS complaints mechanisms will still be available to you (if appropriate).

Thank you for taking the time to read this information sheet**. If you would like to know more, please contact** <<insert contact details>>
